# Biomechanical properties of fishing lines of the glowworm *Arachnocampa luminosa* (Diptera; Keroplatidae)

**DOI:** 10.1038/s41598-019-39098-1

**Published:** 2019-02-28

**Authors:** Janek von Byern, Pete Chandler, David Merritt, Wolfram Adlassnig, Ian Stringer, Victor Benno Meyer-Rochow, Alexander Kovalev, Victoria Dorrer, Simone Dimartino, Martina Marchetti-Deschmann, Stanislav Gorb

**Affiliations:** 1 Ludwig Boltzmann Institute for Experimental and Clinical Traumatology, Austrian Cluster for Tissue Regeneration, Vienna, Austria; 2 University of Vienna, Faculty of Life Science, Core Facility Cell Imaging & Ultrastructure Research, Vienna, Austria; 3 Spellbound Cave, Waitomo, New Zealand; 4 The University of Queensland, Faculty of Science, School of Biological Sciences, Brisbane, Queensland Australia; 5 Department of Conservation, Wellington, New Zealand; 6 Hachijojima: Research Institute of Luminous Organisms, Tokyo, Japan; 7 Kiel University, Zoological Institute, Functional Morphology and Biomechanics, Kiel, Germany; 8 Vienna University of Technology, Institute of Chemical Technologies and Analytics, Vienna, Austria; 9 The University of Edinburgh, School of Engineering, Institute for Bioengineering, Edinburgh, United Kingdom

## Abstract

Animals use adhesive secretions in highly diverse ways, such as for settlement, egg anchorage, mating, active or passive defence, etc. One of the most interesting functions is the use of bioadhesives to capture prey, as the bonding has to be performed within milliseconds and often under unfavourable conditions. While much is understood about the adhesive and biomechanical properties of the threads of other hunters such as spiders, barely anything is documented about those of the New Zealand glowworm *Arachnocampa luminosa*. We analysed tensile properties of the fishing lines of the New Zealand glowworm *Arachnocampa luminosa* under natural and dry conditions and measured their adhesion energy to different surfaces. The capture system of *A*. *luminosa* is highly adapted to the prevailing conditions (13–15 °C, relative humidity of 98%) whereby the wet fishing lines only show a bonding ability at high relative humidity (>80%) with a mean adhesive energy from 20–45 N/m and a stronger adhesion to polar surfaces. Wet threads show a slightly higher breaking strain value than dried threads, whereas the tensile strength of wet threads was much lower. The analyses show that breaking stress and strain values in *Arachnocampa luminosa* were very low in comparison to related *Arachnocampa* species and spider silk threads but exhibit much higher adhesion energy values. While the mechanical differences between the threads of various *Arachnocampa* species might be consequence of the different sampling and handling of the threads prior to the tests, differences to spiders could be explained by habitat differences and differences in the material ultrastructure. Orb web spiders produce viscid silk consisting of β-pleated sheets, whereas *Arachnocampa* has cross-β–sheet crystallites within its silk. As a functional explanation, the low tear strength for *A*. *luminosa* comprises a safety mechanism and ensures the entire nest is not pulled down by prey which is too heavy.

## Introduction

Similar to other groups of animals, insects use adhesives widely in their daily life. Its purposes are diverse and include attachment to surfaces, resistance against external detachment forces (e.g. wind gusts), defence against predators, egg anchorage to oviposition sites, prey capture, etc. (see review)^1^. Much is known about the orb web spiders ecribellates’ prey capture system^2–5^ using viscid threads, the latter produced by flagelliform glands, and coated with secretions from the aggregate glands^6–9^. Yet, the attachment threads for prey capture of other insects remain largely unexplored.

Glowworms are the larval luminescent stage of certain species of keroplatid flies. The genus *Arachnocampa*^10^, contains the world-renowned “glow-worms” endemic to New Zealand and Australia^6,11–14^ and known for its members’ ability to capture prey by means of adhesive threads in combination with bioluminescent lures^13,15,16^.

*Arachnocampa* only lives where the humidity is high and there is low air movement such as sheltered banks in rainforest or tree-fern lined gullies and caves with streams or rivers entering them. The world-famous Waitomo Glowworm Caves and Spellbound Cave on the North Island of New Zealand have populations of thousands of individuals, providing a spectacular display of glowworm bioluminescence day and night.

The larvae construct a nest composed of a mucous tube or ‘hammock” that hangs beneath a solid substrate attached by a network of threads^17^. Long threads, fishing lines, with evenly-spaced adhesive droplets^14^ hang down from the attachment threads to form an adhesive curtain^18,19^ similar in function to a spiders’ web^20^. The larvae use a “sit-and-lure” predatory strategy, catching positively phototactic flying insects with bioluminescence light (peak emission wavelength at 488 nm)^21–24^. This bluish light is emitted from a posterior light organ comprising the modified distal ends of four Malpighian tubules^13,14,25–28^ which project the light downwards. The prey consists of small flying insects (moths, mayflies, sand- & stoneflies, or other *A*. *luminosa* adults) as well as crawling invertebrates (isopods, ants, amphipods, millipedes, or even small land snails) that fall or jump into the fishing lines^29^. A midge, *Anatopynia debilis* (=*Tanypus debilis*, Chironomidae), seems to be the main food source for glowworms in the Waitomo caves^17^. Larvae of this midge live in the mud banks and streams within the caves and are present at high density from October to December after dawn^17^. Other larger insects, such as Hymenoptera (bees and wasps) and Coleoptera (beetles), are caught occasionally in the fishing lines^25^.

Earlier studies have shown that the capture thread system of *Arachnocampa* differs from that of orb web spiders. The *Arachnocampa* fishing line is composed of cross-β-sheet-rich silk^30,31^, while threads of spider webs consists of β-pleated protein sheets forming a “β-spiral” nanospring^32^. The adhesive droplets of spider webs comprise (i) a small central granule, (ii) a larger glycoprotein glue region and (iii) a fluid outer layer consisting mostly of water. In contrast, the droplets on *A*. *luminosa* fishing lines have a two-layered structure with (i) a central core attached to the silk thread, containing a variety of components and salts including urea, and (ii) are surrounded by a relatively thick layer of water^17^. Earlier tests have shown that most of the outer water shell evaporates at a humidity below 80%, leaving only a thin layer of water covering the central core. The dry droplet has then an irregular layer of “salt” crystals surrounding the central core. If a dehydrated droplet is exposed to high humidity it rehydrates to form a complete entity^17^.

*Ex situ* studies of the *Arachnocampa tasmaniensis* fishing lines indicate that beside the adhesive fluid changes the silk threads also change their mechanical properties from high to low humidity conditions. Under dry conditions (<30%RH), the threads exhibit a higher Young’s modulus (18.38 GPa), while the breaking strain (from 0.02 to 0.6 ln (mm mm^−1^)) and toughness (from 2.4 to 25.82 MJ m^−3^) both increase, when tested under high humid conditions (>90%RH)^33^. Unfortunely, in this study the authors used long-term stored dried thread fragments (1 cm length), which were exposed to high humidity conditions just prior to mechanical tests^33^.

To avoid any mechanical and molecular alteration of the fishing lines and their adhesive droplets due to long storage at low humidity, we aimed at investigating the tensile and strain properties of the *Arachnocampa luminosa* fishing lines under *in situ* (within the cave) conditions. Additionally, *ex situ* studies were done near the cave within 24 h of thread collection, with threads stored in defined humid conditions and with minimal external influence during transport. We use analyses of peeling strength to quantify the bonding strength of adhesive droplets, on different artificial and natural surfaces and provide insights into adhesive properties of the capture system.

## Material and Methods

All collections and measurements other than the thread length observations were carried out from October to December 2014 within the Spellbound Cave (S38°19′549; E175°04′454), Waitomo, New Zealand^17^. This is a commercial tourist cave with a wide and easily-accessible entrance and with a large open stream that enters it.

Tensile and peeling strength measurements were taken using newly collected fishing lines in the cave (temperature 13–15 °C, relative humidity RH = 98%) at night after the tourist visits. Measurements on dried threads were taken during daytime in the laboratory (temperature 20 °C, ≈60% RH) in the Waitomo Cave Discovery Centre. Thread length under drying conditions was carried out on *Arachnocampa flava* at the University of Queensland, Australia.

### Instrumental set-up

**Figure 1:**
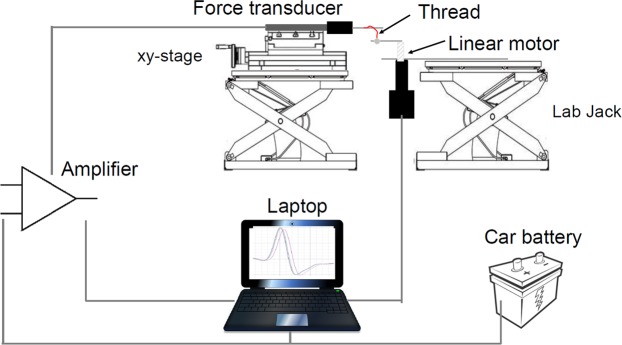
Schematic drawing of the self-constructed test rig and the arrangement of the force transducer, linear motor and data acquisition system. Image drawn by the first author.

We constructed a simple, light-weight apparatus for taking strength measurements of fishing lines (Fig. 1), designed for easy transportation and installation at night in the cave. It was equipped with a 10 gram force transducer (Model FORT 10 g, World Precision Instruments, USA) above a motorized stage (M-168 Linear Actuator Stepper, Physik Instrumente GmbH, Germany). Both instruments were placed on a Swiss Boy lab jack, and the force transducer was mounted on an XY positioning table (KT2304-KL-PT1104, MM Engineering GmbH, Germany). The apparatus was completely enclosed within an acrylic glass cover to protect it against damage during transport and dripping water from the cave ceiling. It also reduced air movement during measurements. Precise alignment of both the apparatus and the force transducer in all directions was verified using a spirit level on the XY table.

Force measurements were made using an MP 100 data acquisition system (BIOPAC Systems Inc., USA), connected to a notebook computer and controlled by the software AcqKnowledge 3.7. A 12 V car battery was used as a power source for the linear motor and the BIOPAC data acquisition system, when taking measurements within the cave. Measurements in the laboratory used mains electricity.

### Instrumental settings and calibration

The signal was fed into the digitizing system using a gain of 200 and acquisition rate was set to 5 kHz. The 10 g force transducer was adjusted with a standard comprising a pre-weighted (750 mg) piece of wire. This was weighed periodically to check that it remained clean and that its mass remained constant. The force transducer was calibrated at both the start, and rechecked and recalibrated at the end of each individual measurement.

### Tensile strength measurements

**Figure 2:**
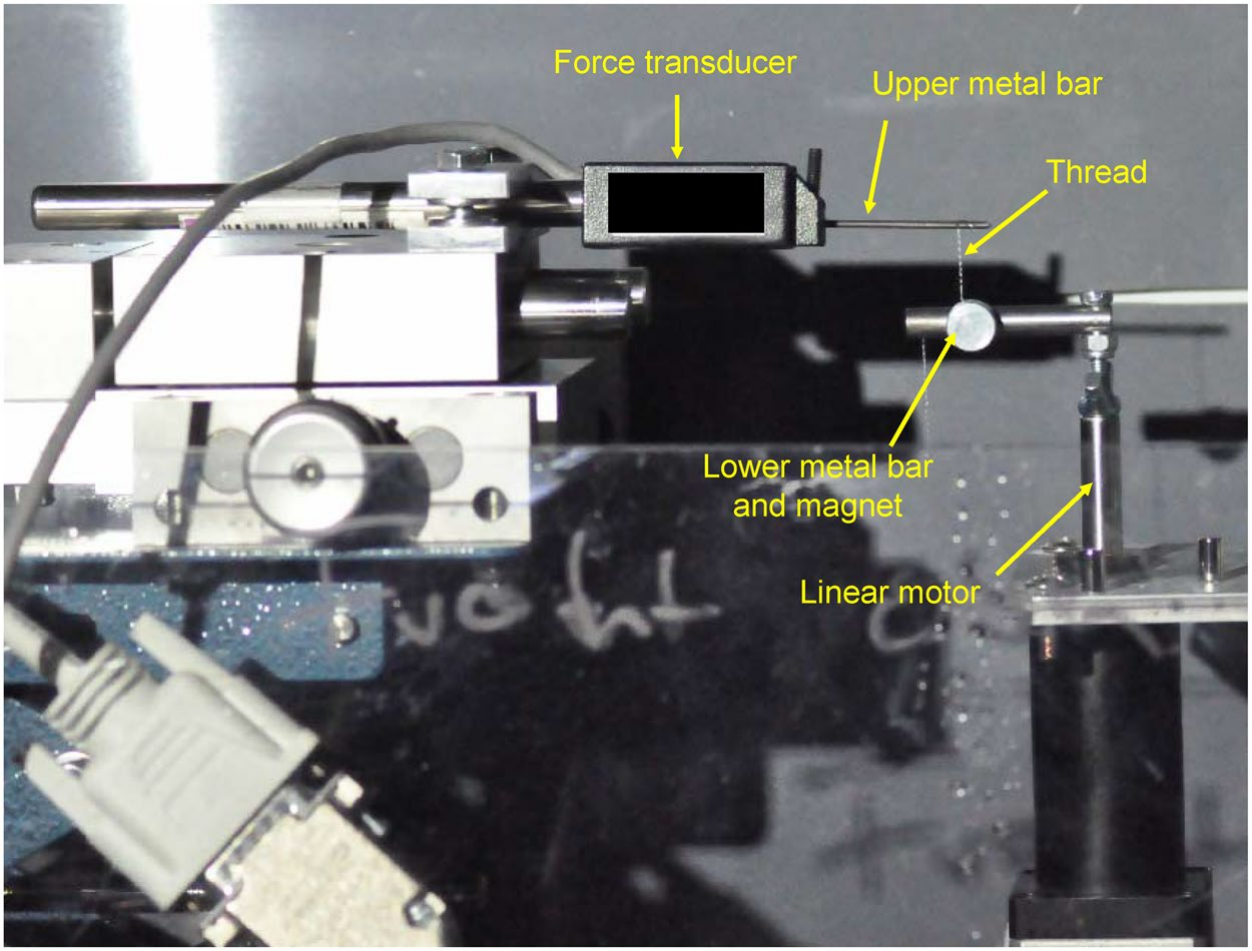
Snapshot of the instrumental set-up and orientation for the tensile strength measurement. The fishing line was wrapped around the upper metal bar and attached to the lower metal bar by a magnet and pulled down with a linear motor at constant speed. Image made by the first author.

Tensile strength tests were conducted with fishing lines fastened between a metal bar (diameter 1.51 mm, length 32.6 mm) which was screwed into the end of the force transducer (Fig. 2) and a thicker steel pin (diameter 3.45 mm, length 11.32 mm) screwed to the linear motor. The distance between the upper bar and lower pin was always set to 15 mm.

Only a single fishing line was collected from a glowworm nest, additional fishing lines were always collected from different nests. This minimized damage to nests and provided better statistical replicates than using multiple fishing lines from the same nest. Long single threads were detached from glowworm nests without destroying the hammocks and fastened to the transducer bar by wrapping them around it several times. The threads were then attached to the lower pin, by wrapping them around it and securing with a magnet. On the upper wire, no magnet was attached on the threads as the magnet appeared to be too heavy for the analyses. When measurements were taken using three fishing lines (subsequently named “triplets”) of the same length and from the same nest, they were twisted by slow rotation around their axis and then fixed as bundle within the instrument as described above for the single thread.

Once fixed to the apparatus, tensile measurements were made after 30 s to allow the transducer and the threads to stabilize, then the lower pin was locked to the motor which then pulled at a constant speed of 0.1 mm/s until the thread broke off. The strain on the threads was calculated after recording the position of the motor at the start and when the threads broke. The transducer wire and motor pin were carefully cleaned with paper towels before new threads were attached.

### Peeling strength measurements

**Figure 3:**
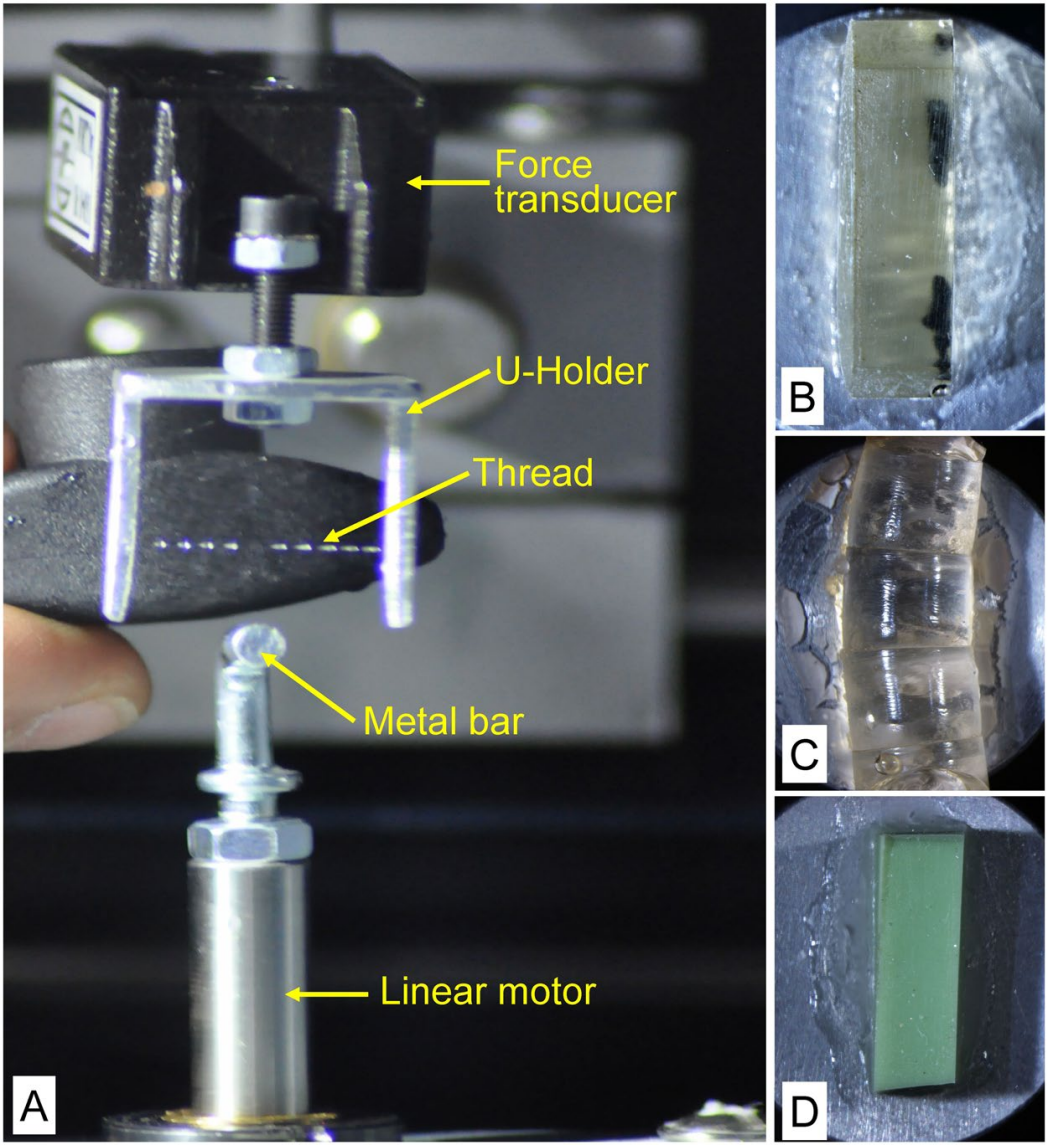
Arrangement of the thread in the u-shaped holder for the adhesion strength measurement. The linear motor with different probe types monted on the top: (**A**) metal bar, (**B**) resin block, (**C**) *Zophobas* mold , (**D**)  silicone block. The motor was first approached to the thread and after contact formation pulled down. Image (**A**) was taken after the measurement, showing a gap in the droplet arragement due to the previous contact with the metal bar.

Peeling strength was measured using the same force transducer and motorized stage as above, except that a u-shaped aluminium frame was attached to the transducer. A 16 mm gap in the frame allowed a probe to make contact with fishing lines when laid across the gap (Fig. 3A). Five different probes were used comprising: (*i*) a stainless steel metal bar (∅ 3.5 mm), (*ii*) a plain, epoxy resin block 3.4 mm wide (Agar 100 Resin, Agar Scientific, UK) (Fig. 3B), (*iii*) an epoxy-cast mold of the abdomen of the larval *Zophobas* (Coleoptera, Tenebrionidae) (Fig. 3C), (*iv*) a plain, PVS (polyvinyl siloxane) block 3 mm wide (Coltene/Whaledent AG, Switzerland) (Fig. 3D) and (*v*) the abdomen of a dead larval *Zophobas*. Each was attached with super glue to a Hitachi screwable stub. These probes were employed one at a time after screwing them into the lower motorized stage.

Newly collected fishing lines (singular or triplet) were wrapped around the two uprights of the frame, then the frame was screwed to the force transducer. Measurements were then taken after 30 s as for tensile testing above. After one of the five different probes were brought into contact with the fishing lines that were orientated horizontally within the u-shaped frame. The probe was then pulled down at a constant speed of 0.1 mm/sec, until contact was broken (Suppl. Video 1). As for the tensile strength measurements, the frame and probes were cleaned with paper towels and distilled water and dried after each measurement.

Each measurement of adhesion strength was simultaneously recorded with a video camera (SONY, Handycam HDR-SR5E). Afterwards still images were extracted from the recorder information with the free software Avidemux Version 2.6.12 were used to measure both thread lengths and contact angle on the different probes shortly before detachment, using the microscopic software cell D (Version 5.1, Olympus, Austria). The size of the u-shaped frame was used in all still images as a reference.

### Tensile and peeling strength tests with dry threads

**Figure 4:**
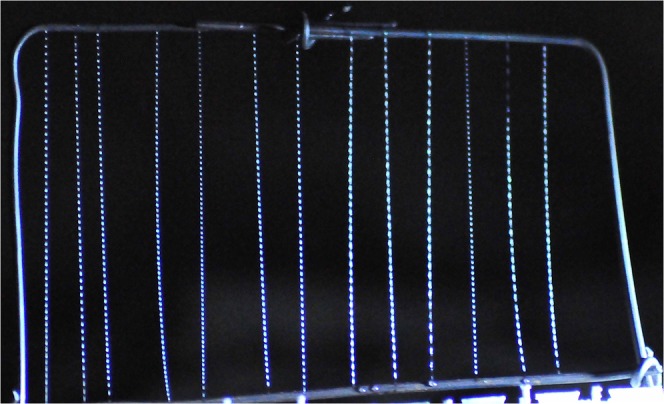
For drying the fishing lines, each thread was individually attached to a metal frame, deposited in a desiccator filled with a drying agent and transported outside the cave. After 24 h, the threads were carefully removed and attached to the metal bars for the tensile strength measurements.

Single or triplet fishing lines were collected as above for both tensile and peeling strength tests and fixed to the same wire or frame as above (Fig. 4). They were then placed in a desiccation cabinet at 24 °C and 60% RH filled with dry calcium chloride for a minimum of 24 h. Measurements of tensile and peeling strength was made as above in the laboratory (24 °C, 60% RH) immediately after removing the threads from the desiccation cabinet.

### Thread length under desiccation

Larvae of *Arachnocampa flava* taken from the field were kept in the laboratory in artificial habitats carved from moistened phenolic foam blocks (floral foam) and stored in an aquarium with water covering the base. Individual threads were detached from a larval snare using fine forceps and mounted on a stand placed in a glass aquarium. The thread was imaged using a Sony A7S camera and 100 mm Canon macro lens, taking a photograph (4240 × 2384 pixels) every 20 sec. A ruler was photographed after each run to provide a scale reference. As soon as the thread was installed, the aquarium was sealed and dry ambient air was introduced into the aquarium through a plastic tube terminating in a bubbler stone to reduce turbulence. The air was dried by pumping it through a chamber filled with silica gel desiccating beads. For analysis, thread length was measured from the individual images using ImageJ.

### Statistical evaluation

Statistical evaluation of all peeling strength data and thread strength data were performed in MS Excel and Stata 14.2 and presented as boxplot graphs.

The skewness-curtosis test was used to ensure that the data did not differ significantly from normal distributions (P ≥ 0.05) before further analyses. Differences between two or multiple samples were tested with the unpaired t-test and one-way ANOVA tests followed by the post hoc Bonferroni test were used for multiple samples, P < 0.05 was regarded as significant, P < 0.01 as highly significant.

Calculation of the tensile strength were made using a mean thread diameter of 8 µm based on morphological data^17^ and a local gravity value of 9.80 m/s^2^ for Waitomo, New Zealand (based on the Gravity Information System of the Physikalisch-Technische Bundesanstalt Germany; https://www.ptb.de/cartoweb3/SISproject.php).

### Measuring surface wettability of probes

The polar and non-polar components of the surface energy of all five probes (metal bar, resin block, epoxy *Zophobas* larva mould, PVS block, dead *Zophobas* larva), was measured using the ‘sessile drop’ method^34^ with a OCAH 200 high-speed contact angle measuring system (DataPhysics Instruments GmbH, Filderstadt, Germany) equipped with silanized glass capillaries (tip diameter 0.15 mm) and two polar (distilled water and ethylene glycol) and one non-polar liquid (diiodomethane). Each measurement was taken after depositing 2 µl of one of the three fluids onto the probe and taking an image of the droplet once its shape had stablized. This was repeated at least five times (Table 1) with the contact angle being measured using the software SCA20 (DataPhysics Instruments). Measurement were taken under ambient conditions (25 °C and 60% RH).

**Table 1:** Surface energy of different surfaces used in this study and contact angle data of the polar and dispersive liquids.

	Surface Free Energy [mN/m]	Dispersive [mN/m]	Polar [mN/m]	Water [Θ]	Ethylene glycol [Θ]	Diidomethane [Θ]
Metal bar	28.34	26.82	1.52	93.7 ± 3.1 SE	79.5 ± 2.1 SE	49.3 ± 6.4 SE
Epoxy resin block	25.87	20.65	5.21	86.8 ± 1.7 SE	19.0 ± 2.2 SE	59.2 ± 3.4 SE
*Epoxy Zophobas* abdomen mold	32.98	26.24	6.73	78.7 ± 4.9 SE	63.9 ± 3.8 SE	44.9 ± 4.6 SE
PVS block	27.17	12.05	15.11	73.2 ± 5.9 SE	82.2 ± 1.1 SE	62.8 ± 2.5 SE
*Native Zophobas abdomen*	34.49	32.90	1.59	90.8 ± 2.8 SE	65.4 ± 4.2 SE	42.9 ± 3.9 SE
Glass (control)	65.86	12.41	53.45	20.5 ± 1.7 SE	15.4 ± 2.6 SE	56.5 ± 0.6 SE

### Adhesion energy estimation

**Figure 5:**
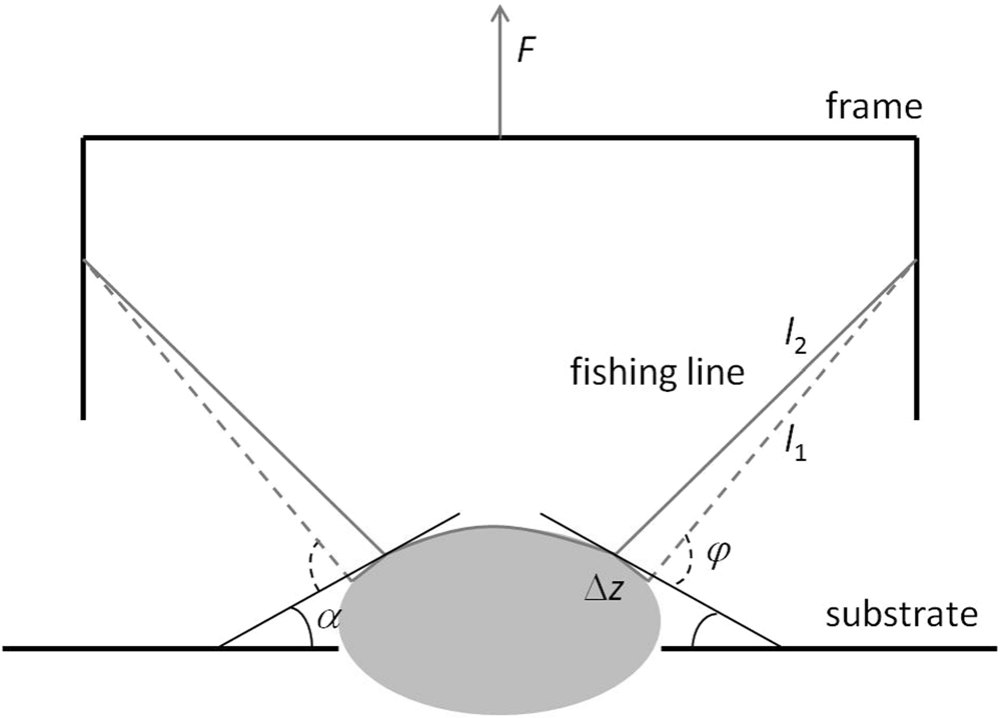
Scheme of the peeling experiment. The probe is shown as a gray ellipse. The fishing line is shown as a gray line. *F* is the frame pulling force, *φ* is a peeling angle, *α* is the sample slope at the pull off. At pull off a small section of the fishing line with the length Δz is releasing from the sample, and the length of the free fishing line changes from *l*_1_ to *l*_2_.

The adhesion energy in the peeling experiments was estimated from the energy balance at detachment of an incompressible neo-Hookean thread from a substrate in a way similar to that previously used by Kendall^35^. The line pull-off from the substrate takes place when the energy balance at its infinitesimal release from substrate Δ*z*, (Fig. 5), is negative:

1 $${\rm{\Delta }}{E}_{el}-{\rm{\Delta }}{E}_{s} < 0,$$

where *E*_el_ and *E*_s_ are elastic and surface energies. We assume: ${\rm{\Delta }}{E}_{s}=-\,Wd{\rm{\Delta }}z,$ where *W* is the adhesion energy, *d* is the contact width (it was assumed to be equal to the fishing line diameter, 8 µm). The elastic energy of a neo-Hookean thread was taken equal to Odgen^36^:

2 $${E}_{el}=\frac{\mu {A}_{0}{l}_{0}}{2}({\lambda }^{2}+2{\lambda }^{-1}-3),$$

where *μ* is a Lamé coefficient (shear modulus), *l*_0_ is the initial length, *A*_0_ is the initial cross-section area. For the extension ratio *λ*, under assumption of an infinite friction between the thread and substrate, for experimental scheme shown in Fig. 5 we can write:

3 $${\lambda }_{1}={l}_{1}/{l}_{0},{\lambda }_{2}^{2}=({l}_{1}^{2}+{\rm{\Delta }}{z}^{2}+2{l}_{1}{\rm{\Delta }}z\,\cos (\phi ))/{({l}_{0}+{\rm{\Delta }}z)}^{2},$$

where *φ* is a peeling angle. The change in elastic energy at infinitesimal pull-off is then given by ${\rm{\Delta }}{E}_{el}=\frac{\mu {A}_{0}{l}_{0}}{2}({\lambda }_{2}^{2}+2{\lambda }_{2}^{-1}-{\lambda }_{1}^{2}-2{\lambda }_{1}^{-1})$. Taking into account Eq. 3 for Δ*z* → 0, neglecting the terms with Δ*z*^k^ for k > 1, after simplification, one obtains: ${\rm{\Delta }}{E}_{el}=-\,\mu {A}_{0}{\lambda }_{1}{\rm{\Delta }}z(1-{\lambda }_{1}^{-3})({\lambda }_{1}-\,\cos (\phi ))$. The frame pulling force equals to: $F=2T\,\sin (\phi -\alpha ),$ where *α* is a substrate slope at the peeling position and the traction force equals to Odgen^36^: $T=\mu {A}_{0}(1-{\lambda }^{-3})$. So, ${\rm{\Delta }}{E}_{el}=-\,\frac{F\lambda (\lambda -\,\cos (\phi )){\rm{\Delta }}z}{2\,\sin (\phi -\alpha )}$, and from ${\rm{\Delta }}{E}_{el}={\rm{\Delta }}{E}_{s}$ we obtain the following expression for the adhesion energy:

4 $$W=\frac{F\lambda (\lambda -\,\cos (\phi ))}{2d\,\sin (\phi -\alpha )}$$

### Additional data

Additionally, three individuals of *Arachnocampa luminosa* (one with a length around 20 mm and two with a length of 40 mm) and 100 individuals of the midge *Anatopynia debilis*, the main food source of *Arachnocampa luminosa* in Spellbound, were collected and its wet weight measured individually.

## Results

### Strain

**Figure 6:**
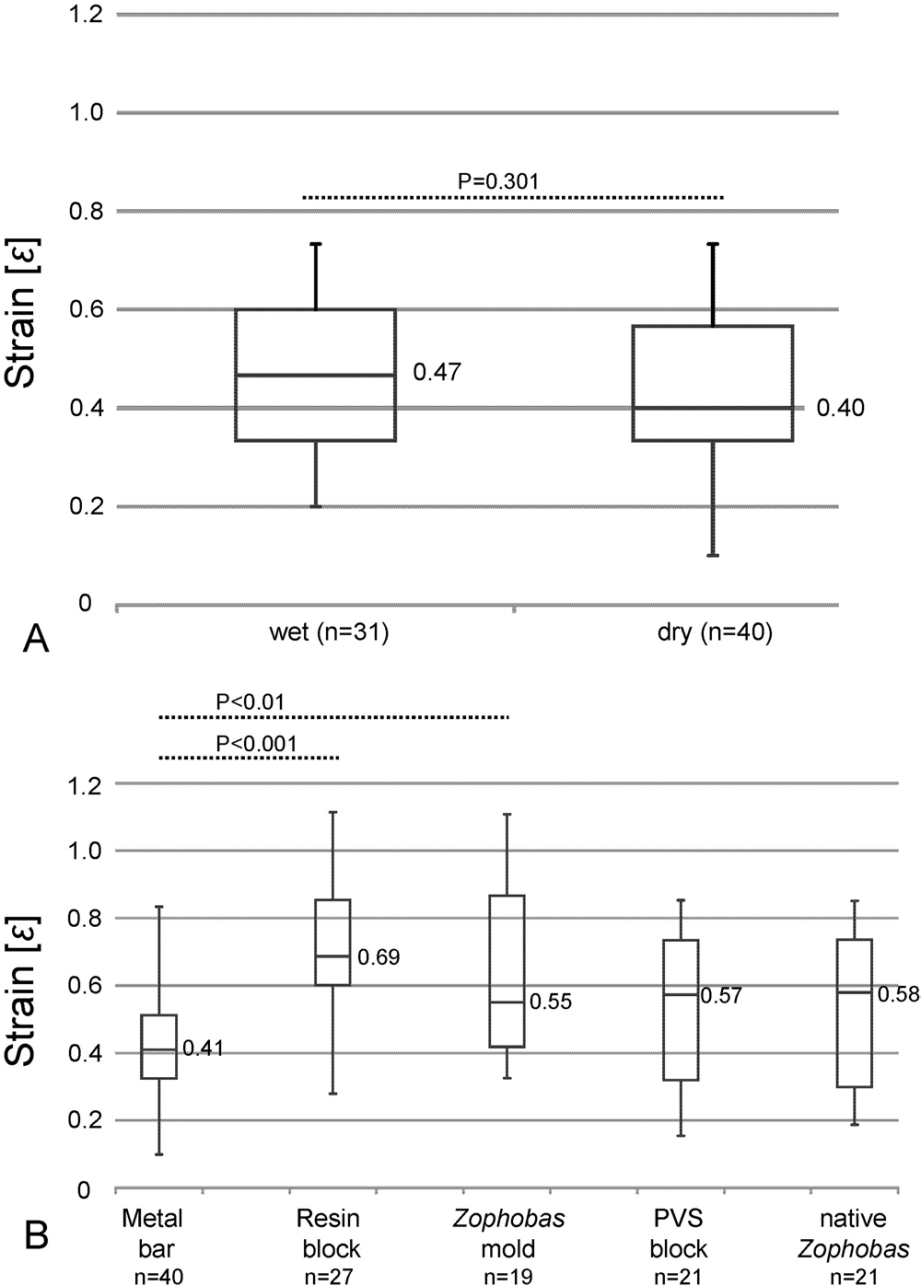
Boxplot chart of the strain values of the *Arachnocampa* fishing lines, the top and bottom of the boxes indicate 75 and 25 percentiles, the values are medians. (**A**) Vertically-pulled wet threads (measured at 100% RH in the cave) show no significanct difference (P = 0.301) in relation to dried ones (incubated at 60% RH, 20 °C). (**B**) Extensibility analyses of inclined wet threads point out for most probe types slightly higher strain values (0.55–0.69; *Zophobas* larva mold → resin block). Only the threads measured in contact with the metal bar show slightly lower strain values (0.41). Significant differences could be observed between the experiments on the metal bar and resin block (P < 0.001) as well as metal bar and *Zophobas* larva mold (P < 0.01) only (see Table S1 for details).

Newly collected fishing lines tend to have not statistically significant higher strain (0.47) than dry ones (0.40) when pulled vertically during the tensile strength tests (Fig. 6A). In peeling experiments, wet threads show slightly, but not significantly higher, extensibility on all probes (0.55–0.69) than on metal bar (0.41) (Fig. 6B). Statistically significant differences could only be observed between the stainless steel metal bar and the resin block or the epoxy *Zophobas* larva mold (Table S1).

### Tensile strength

Preliminary tests showed that newly collected fishing lines did not adhere well to the metal bar and had to be wrapped several times around it to ensure reasonable “clamping”. However, these lines could not be wrapped around the lower bar without stretching them unintentionally so they were anchored using a magnet.

**Figure 7:**
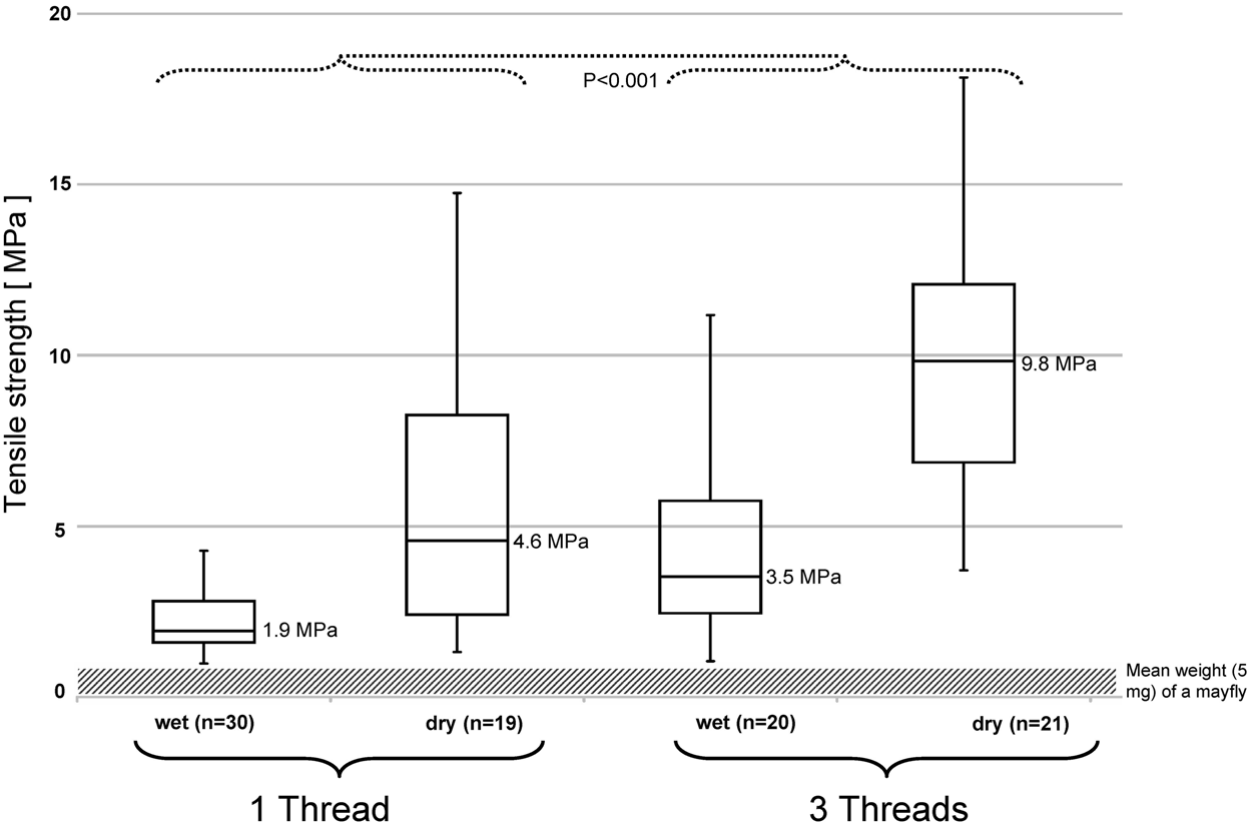
Boxplot diagram of the tensile strength values of *Arachnocampa* fishing lines. In general, threads (singular or triplet arranged) dried in the desiccator cabinet (60% RH, 20 °C) display higher tensile strength values than the wet ones, however show no bonding ability. The single wet threads are able to hold the load up to two average prey items (mean weight of *Anatopynia debilis* = 5 mg), while more/or heavier preys could be held by tangled up threads. Differences between tensile strength for the triple-arranged threads (wet/dry) and the single ones (wet/dry) are highly significant (P < 0.001).

Measurements with single newly collected fishing lines gave a tensile strength of 1.9 MPa (maximum force = 0.1 mN) (Fig. 7) that should be sufficient to catch and support one or more prey items as *Anatopynia debilis* (weight between 1 and 10 mg). Although not tested in this study, it could be assumed that the thread type used to attach the “hammock” to the rocky substrate displays higher tensile strength values than the fishing lines used for prey capture. Weight measurements yield for the 20 mm long larva 30 mg and for the larger, 40 mm long ones a weight of 100 mg, ten times heavier than the maximum load derived from the tensile strength values for a single fishing line.

Triplet-combined fishing lines only doubled the tensile strength to 3.5 MPa (maximum force = 0.17 mN), indicating that these lines interact slightly and do not break simultaneously. However, as the moving prey during catch often comes in contact with several fishing lines and tangles them up, this multi-thread effect would provide weight relief for each thread and support larger or more prey items.

**Figure 8:**
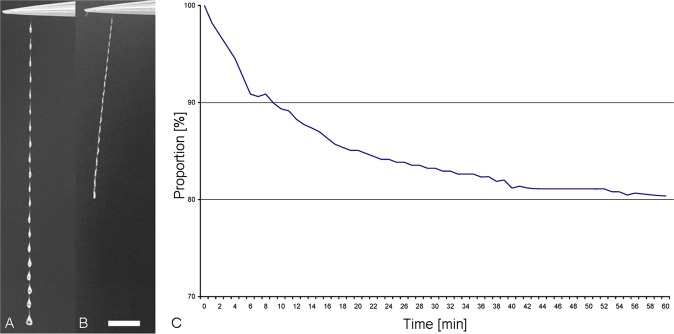
Fishing line of *Arachnocampa flava* at (**A**) start and (**B**) after 60 min of dry air exposure. Beyond the thread shortening also a droplet mass loss could be observed. (**C**) Time-course analyses of the thread shortening during dry air exposure. Scale bar for A and B = 2 mm.

Time-lapse imaging of single threads freshly collected (Fig. 8A) and dried (Fig. 8B) showing that the lines shorten and the droplets leave a residue as they evaporate. When threads were removed from a snare and immediately exposed to dry air, they shortened to approx. 80% after 60 min and 59.1% of their original lengths after 100 min, respectively (Fig. 8C). As the threads dry and lose droplet mass, they tend to drift in the slightest air movement and could possibly become statically charged.

**Figure 9:**
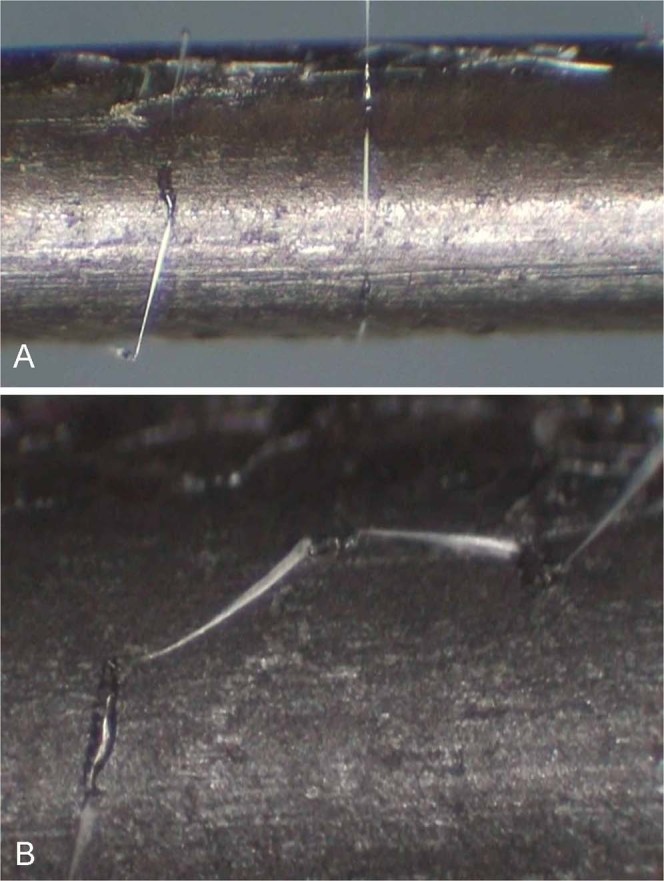
(**A**) Attachment of the dried fishing line to the metal bar for the tensile strength measurements. (**B**) Higher magnification shows that the droplet core (yellow arrow) firmly attached to the metal when loaded, while the silk thread (yellow arrowhead) shows no sign of bonding.

Dried fishing lines have a much higher tensile strength than wet ones, e.g., 4.6 MPa (maximum force = 0.23 mN) for single fishing lines and 9.8 MPa (maximum force = 0.49 mN) for three combined (P < 0.001) (Fig. 7B). Moreover, drying of the fishing lines under low humidity (<80% RH) resulted in dehydration of the droplet^17^ and only a small remnant comprising the central core remained visible. The dried fishing lines also appeared stiffer and could not be smoothly wrapped around the upper bar in the same way as fresh threads. The silk thread of dry fishing lines also showed no adhesion to the probe holder, whereas the dry remnants of the droplets attached firmly to the metal surfaces (Fig. 9) of the upper bar and pin under tension.

### Peeling strength

**Figure 10:**
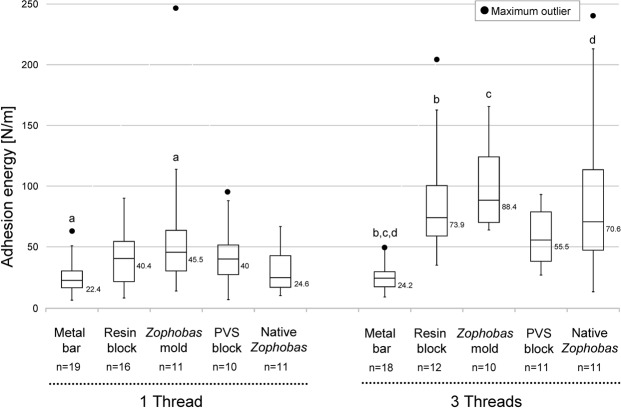
Adhesion energy of wet *Arachnocampa* fishing lines measured on different probe types. The energy of the single thread reaches higher values (40–45 N/m) on polar surfaces (resin block, *Zophobas* larva mold, PVS block) than on metal bar and native *Zophobas* (22–24 N/m), with outliers from 6 N/m up to 248 N/m. In triple-arranged threads, most values are doubled, only on the native *Zophobas* surface, a three times increase from 24.6 N/m (single thread) to 70.5 N/m was observed. A highly significance (see also Supplement Table 2) could be observed for the metal bar and *Zophobas* larva mold (a): P = 0.007), the metal bar and resin block (b): P = 0.001) as well as *Zophobas* larva mold (c): P < 0.001) and native *Zophobas* (d): P = 0.001).

Newly collected single fishing lines showed the same minimum adhesion energy of 6 N/m on different probes (Fig. 10). The epoxy *Zophobas* larva mold displayed the widest range of adhesion energy (up to 113 N/m with an outlier of 248 N/m) compared to the other probes. The adhesion energy on the resin block (40.4 N/m), the epoxy *Zophobas* larva mold (45.5 N/m) and the PVS block (40 N/m) were almost double that of the metal bar (22.4 N/m) and the *Zophobas* larva (24.6 N/m). The low adhesion energy values for the metal bar and *Zophobas* larva could presently not be explained from the surface energy values of those probes (Table S2). Statistically significant differences for the metal bar vs. the Zophobas mold (a: P = 0.007) and the PVS block vs. native *Zophobas* (b: P = 0.062) were observed.

Measurements with triplets of fresh fishing lines resulted in an approximate doubling of the median adhesion energies for the resin block (73.9 N/m), *Zophobas* larva mold (88.4 N/m) and PVS block (55.5 N/m). This result is similar to tensile strength measurements. In contrast, the median adhesion energy on the metal bar was no different between single (22.4 N/m) and three threads (24.2 N/m), while adhesion energy on the *Zophobas* larva probe was about the triple (70.6 N/m) than that for a single fishing line (24.6 N/m). *Zophobas* larva probe also showed the widest range of adhesion energies from 20 N/m to 225 N/m. The calculated adhesion energy was statistically different between the metal bar and resin block (P = 0.001), the metal bar and *Zophobas* larva mold (P < 0.001) as well as the metal bar and native *Zophobas* (P = 0.001) (Table S2).

Peeling strength experiments could not be carried out on dry fishing lines as they showed no adhesion to any of the different probes.

## Discussion

This study provides the first description of the biomechanical properties of the fishing lines of *Arachnocampa luminosa* larvae *in situ* and details on their bonding strength to materials with different surface energies.

The collected fishing line of *A*. *luminosa* displays a slightly, but not significantly, higher extensibility than dry threads; visual observations confirm that dehydration also results in a loss of flexibility, as mentioned previously^17,31,33^.

Comparison with data given for the fishing lines in *Arachnocampa tasmaniensis* show large differences in mechanical properties. The tensile strength in *A*. *tasmaniensis* is much higher (122.77 MPa at 90% RH; 159.04 at 30% RH)^33^ than that measured for *A*. *luminosa* (from 1.9 MPa at RH = 100% to 4.6 MPa at RH = 60%). Also, the extensibility of the fishing lines is slightly higher under highly humid conditions (*A*. *tasmaniensis = *0.6; *A*. *luminosa* = 0.47). Interestingly, at 60% RH, the threads still show an ability for extension (*A*. *luminosa* = 0.40), while at 30% RH it is rather low (*A*. *tasmaniensis* = 0.02)^33^.

The question arises, whether these measured differences are a consequence of the different sampling and handling of the threads prior to the tests. Fishing lines from *A*. *tasmaniensis* were affixed for air transport with wood working glue on a paper frame, stored for a long time under dry conditions and segments of these samples were re-hydrated for a short period at 90% RH before the test series^33^. The authors, however, assure that their sample handling had no influence on the mechanical properties^33^. In the present study in *A*. *luminosa*, in contrast, long freshly-collected segments (>4 cm) of the *A*. *luminosa* fishing lines were measured *in situ* with minimal handling and mechanical stress before the measurement.

However, it could also not be excluded that the silk composition in both species differs, leading to different mechanical properties. The element composition of the fishing line of *A*. *tasmaniensis* seems to contain a higher amount of sulphur beside carbon and calcium in relation to those measured for *Arachnocampa luminosa*^17^ and quantified for *A*. *richardsae*^31^. Disulfide bonds are known to link in *Bombyx mori* silk two different fibroin chains (the hydrophobic heavy and the elastic hydrophilic light chain)^37,38^ necessary to form fibroin in a normal ratio for the cocoon production^37^. Further studies are therefore planned to quantify the amino acid composition in both *Arachnocampa* species in detail and repeat the biomechanical tests in *A*. *tasmaniensis* as done for *A*. *luminosa* to confirm different mechanical properties as a consequence of its molecular composition.

In this regard, the *Arachnocampa* fishing line silk has also very different mechanical properties to the egg stalk threads of the green lacewing *Chrysopa carnea*, those silk also exhibits a cross-β-sheet structure. The egg stalks have much higher extensibility (4–6 at RH = 100% and 2.5 at RH = 65%^39,40^) and tensile strength (232 MPa at RH = 100% to 70 MPa at RH = 30%)^39^ than *A*. *luminosa* (present study) and *Arachnocampa tasmaniensis*^33^, probably because “glow-worm silk is likely to have a substantially lower crystalline fraction, compared to either silkworm silk or lacewing egg-stalks”^31^. However, the differences in the strain and tensile strength between hydrated and dry *C*. *carnea* egg stalks presumes that the increased extensibility and strength is largely influenced by the hydrogen bonds between the cross-β strands which results in a rearrangement of the cross-β strands into a parallel β-sheet conformation^39^ with increasing humidity. In representatives of *Arachnocampa*, the threads are fully saturated with water and deformation is effected by “the amorphous fraction, or due to deformation of both the amorphous fraction and the cross-β-sheet crystallites“^31^. Absence of the hydrogen bond donor may hinder this rearrangement, leading to higher tensile strength and lower extensibility.

Beside *Chrysopa carnea*, the *Arachnocampa* fishing lines also exhibit lower extensibility values than the β-pleated sheet viscid silk of orb web spiders (2.7–5)^41–43^ or mussel byssal threads (distal-proximal 1.1–2)^42–45^ or elastin (1.5)^46^. It is, however, similar to the dry major ampullate silk (0.22–0.5)^42,43,45^ or the single axial fibre of dry capture thread of cribellate spiders (0.3–0.6)^47,48^.

Meyer-Rochow^13^ reported that glowworm fishing lines of the same diameter were capable of supporting a weight of 15 mg before they snapped, which agrees with our measurements. These results indicate that *A*. *luminosa* fishing lines perform poorly in terms of tensile strength. In orb web spiders, a tensile strength up to 1.1 GPa was reported for major ampullate silk and 0.5 GPa for viscid silk^42^. Also, the cocoon silk of *Bombyx mori* (0.6 GPa)^42^ and the byssus thread of mussels (35–75 MPa, proximal to distal)^44^ are much stronger and somewhat tougher. The majority of other biomaterials, such as tendon collagen (0.12 GPa)^49^, bone (0.16 GPa)^42^, or artificial materials, such as nylon fibre (0.95 GPa)^42^, Kevlar (3.6 GPa)^50^ and carbon fibre (4 GPa)^50^, also have higher tensile strength properties than those of *A*. *luminosa* silk.

The reason for such relatively low extensibility and tensile strength of *A*. *luminosa* fishing lines could have a functional explanation, related to their prey and habitat. For example, orb web spiders require a web with high extensibility and energy-absorbance properties, able to resist the impact of any prey hitting the web at high velocity, be it small flies, heavy beetles or grasshoppers, and, at the same time, supporting the mass of the spider^51^. Moreover, spider web also has to withstand rapidly changing climate conditions (rain, winds, humidity and temperature fluctuations, UV-radiation) without breaking^51^. On the other hand, glowworms build their nests directly above rivers in caves (see Fig. 1 in^17^) or under overhangs in sheltered humid environments such as adjacent to streams and these exhibit relatively minor varying climate conditions throughout the year^26^. Their prey comprises a high proportion of small flying dipterans (89%), about 1–4 mm long^25^ and of relatively low mass as reported here for the prey *Anatopynia debilis*. Large heavy arthropod prey is rarely caught by *A*. *luminosa*^25^. As a consequence of the degree of prey specialization, glowworms may not necessarily need silk that is as resilient as those of orb web spiders.

It is also conceivable that the low strain and tensile strength values of the *A*. *luminosa* fishing lines serve as a safety mechanism. The web structure might hold prey that are small enough to be overcome and eaten, whereas larger prey that could destroy the snare and damage the larva readily break free. Also, it may limit the number of prey items collected and their total load, thereby preventing the nest from being detached from the substratum. Glowworms do occasionally fall from nests and survive if they can find an overhang to produce a new nest^13^.

While *A*. *luminosa* fishing line silk shows a low tensile strength compared to other silk-producing animals, its adhesive properties exhibit much higher values than those of *A*. *tasmaniensis*^33^ and for example orb web spiders (Table 2). In the present study, adhesive droplets of *A*. *luminosa* have a mean adhesion energy of 20–45 N/m, while the values for *A*. *tasmaniensis* and different spider species range from 0.02 N/m to 0.1 N/m^33,52–56^. The adhesive droplets of orb web spiders show similar bonding ability at both high (100%) and low (≈60%) relative humidity levels, while bonding in *A*. *luminosa* is highly dependent on humidity levels. Other biological adhesives have weaker (e.g. 2.5–17.5 N/m for the ascidian *Botrylloides* sp.)^57^ or stronger surface energy values (97 N/m for Crucifix Toad *Notaden bennettii*)^58^ than *A*. *luminosa* droplets, but the latter is comparable with some commercial products such as fibrin (20 N/m) and gelatin-resorcinol-formaldehyde (39 N/m)^58^. The bonding abilities of some biological adhesives also need to be treated with caution depending on the substrates the adhesives were tested with (e.g. sandpaper, glass, animal tissue) as the substrates can greatly influence adhesion energy.

**Table 2:** Adhesion energy values of different biological and commercial adhesives depending on probe type and relative humidity.

Order	Species	Probe type	Relative humidity [%]	Peeling strength [N/m]*	Reference
Diptera	*Arachnocampa luminosa*	Metal bar/native *Zophobas*	100	22.4/24.6	Present study
		Resin block/*Zophobas* larva mold/PVS block		40.4/45.5/50	
		Metal bar/native *Zophobas*	60	0/0	
		Resin block/*Zophobas* larva mold/PVS block		0/0/0	
	*Arachnocampa tasmaniensis*	Glass slide	>90^#^	0.02 (=23.66 µN/mm)	^33^
			30	0.0002 (=0.25 µN/mm)	
Araneae	*Cyrtarachne* sp.	Sandpaper (grit size #1000)	100	0.04–0.1 (≅40–100 µN/mm)	^52^
			60	0.01–0.02 (≅10–20 µN/mm)	
	*Larinia argiopiformis*		100	0.01–0.04 (≅10–40 µN/mm)	
			60	0.01–0.03 (≅10–30 µN/mm)	
	*Neolana pallida*	Sandpaper (grit size #320)	70	0.00552 (≅5.52 µN/mm)	^53– 56^
	*Miagrammopes animotus*		62	0.03150 (≅31.50 µN/mm)	
	*Cyclosa conica*		61	0.01147 (≅11.47 µN/mm)	
	*Leucauge venusta*		61	0.0193 (≅19.3 µN/mm)	
	*Araneus marmoreus*		61	0.03476 (≅34.76 µN/mm)	
	*Larinioides cornutus*	Glass slide	15/80/90	≈4 × 10^−7^ (≅0.4 µJ)/≈8 × 10^–7^ (≅0.8 µJ) µJ/≈4 × 10^−7^ (≅0.4 µJ)	^57, 65^
Ascidian	*Botrylloides* sp.	Mussel shell, eelgrass	100	2.5–17.5	^57, 58^
Amphibia	*Notaden bennettii*	Human cartilage (Meniscus)	≥80	97	^58^
Commercial adhesives	Fibrin	Human cartilage (Meniscus)	≥80	20	^58^
	Gelatin-resorcinol-formaldehyde (GRF)			39	
	n-Bu-2-cyanoacrylate			149	
	Polyacrylamide hydrogel	Mice tissue (heart, lung, kidney, bone)	n.d.	40	^66^
	Modified polyacrylamide hydrogel			166–780	

^*^Orginial values in normal brackets.

^#^re-hydration after storage at low humid conditions.

The present investigation confirms an earlier observation that high humidity (>80% RH) is essential for *A*. *luminosa* to catch preys with its fishing lines^17^. Low humidity not only affects the properties of the silk fishing lines, but it also leads to dehydration of the adhesive droplets even when re-hydrated as in the case of *A*. *tasmaniensis*^33^. Such water loss, (water normally comprises 98% of droplets)^43^ reduces the bonding ability of the glue, making it impossible to catch prey. Adhesion by the central droplet core after dehydration, which is presumably a gluing component, only becomes possible when the fishing line is under artificially produced tension. Under desiccating conditions, threads shorten and wave about more readily, whereas wet lines with large adhesive droplets are more resistant to tangling and maintain their sheet-like spacing. The droplets, however, are hygroscopic and can rehydrate at high humidity^17^. Thus, the prey capture system of *A*. *luminosa* can recover after a dry spell to a small extent as indicated with *A*. *tasmaniensis*^33^, but maybe not to its initial strength. Together, the characteristics of the threads and droplets indicate that *Arachnocampa’s* capture system is highly specialised to their habitat. As a consequence, we suggest that the humidity levels in glowworm tourist caves be maintained above 80%.

The highly-aqueous glue (water content up to 80%) of orb web spiders, is also hygroscopic^59,60^ and can absorb water from the humid atmosphere. In this case, however, the water content does not affect significantly the adhesion strength^61,62^: the glue does not loose its adhesive properties even at a relative humidity of 20%^59,60^. Other environmental factors, such as ultraviolet radiation or temperature^62,63^, have minor effects on the glue of orb web spider, while nothing is yet known in *A*. *luminosa*. Arrangement of the *A*. *luminosa* fishing line droplets and chemical glue composition differ greatly from that of orb web spiders^17^. Such differences can be explained by the different environments these species strive, dictating appropriate evolution of different adhesive systems. The present study shows that capture systems of orb web spiders and *A*. *luminosa* clearly also differ biomechanically so the genus *Arachnocampa* still remains only a superficial reference to the “spider-like habit of the larva, forming webs and using them for the capture of insect prey”^64^.

## Supplementary information

Tables S1 and S2

Supplementary Video 1
