# Supplementary material for: Biomechanical properties of fishing lines of the glowworm Arachnocampa luminosa (Diptera; Keroplatidae)
Source: Sci Rep. 2019 Feb 28;9:3082. doi: 10.1038/s41598-019-39098-1 (PMC6395680; doi:10.1038/s41598-019-39098-1)
Supplement: Supplementary file 1 — Tables S1 and S2 [file 41598_2019_39098_MOESM1_ESM.pdf]

Title

Biomechanical properties of fishing lines of the glowworm *Arachnocampa luminosa* (Diptera; Keroplatidae)

Authors

Janek von Byern[1, 2 *], Pete Chandler[3], David Merritt[4], Wolfram Adlassnig[2], Ian Stringer[5], Victor Benno Meyer-Rochow[6], Alexander Kovalev[7], Victoria Dorrer[8], Simone Dimartino[9], Martina Marchetti-Deschmann[8], Stanislav Gorb[7]

Addresses

[1] Ludwig Boltzmann Institute for Experimental and Clinical Traumatology, Austrian Cluster for Tissue Regeneration, Vienna, Austria; [2] University of Vienna, Faculty of Life Science, Core Facility Cell Imaging & Ultrastructure Research, Vienna, Austria; [3] Spellbound Cave, Waitomo, New Zealand; [4] The University of Queensland, Faculty of Science, School of Biological Sciences, Brisbane, Queensland, Australia; [5] Department of Conservation, Wellington, New Zealand; [6] Hachijojima: Research Institute of Luminous Organisms, Tokyo, Japan; [7] Kiel University, Zoological Institute, Functional Morphology and Biomechanics, Kiel, Germany; [8] Vienna University of Technology, Institute of Chemical Technologies and Analytics, Vienna, Austria ; [9] The University of Edinburgh, School of Engineering, Institute for Bioengineering, Edinburgh, United Kingdom

Supplement data

Table

Table S1: Significance values (*Post hoc* Dunn test) of comparative differences shown by e the elasticity of *Arachnocampa* fishing lines adhering to different materials. Highly significant values are highlighted in bold.

	Resin block	*Zophobas* larva mold	PVS block	*Zophobas* larva
Metal bar	**0.001**	**0.004**	0.387	0.347
Resin block	-	1.000	0.069	0.078
Zophobas larva mold	-	-	1.000	1.000
PVS block	-	-	-	1.000

Table S2: Significance values (*Post hoc* Dunn test) of the *Arachnocampa* adhesion energy on different probe holders. Significant values are highlighted in bold. The P value of the one-factorial Anova was > 0.001 for both tables.

1 Thread

	Resin block	*Zophobas* larva mold	PVS block	*Zophobas* larva
Metal bar	1.000	**0.007**	1.000	1.000
Resin block	-	0.185	1.000	1.000
Zophobas larva mold	0.185	-	0.512	0.062
PVS block	1.000	0.512	-	1.000

3 Threads

	Resin block	*Zophobas* larva mold	PVS block	*Zophobas* larva
Metal bar	**0.001**	**< 0.001**	0.276	**0.001**
Resin block	-	1.000	0.732	1.000
Zophobas larva mold	1.000	-	0.185	1.000
PVS block	0.732	0.185	-	0.719

Video

Supplementary Video 1: Full time video of a peeling strength test with a wet thread in the cave.
